# Perfluorooctanoic Acid (PFOA) Exposures and Incident Cancers among Adults Living Near a Chemical Plant

**DOI:** 10.1289/ehp.1306615

**Published:** 2013-09-05

**Authors:** Vaughn Barry, Andrea Winquist, Kyle Steenland

**Affiliations:** Rollins School of Public Health, Emory University, Atlanta, Georgia, USA

## Abstract

Background: Perfluorooctanoic acid (PFOA) is a synthetic chemical ubiquitous in the serum of U.S. residents. It causes liver, testicular, and pancreatic tumors in rats. Human studies are sparse.

Objective: We examined cancer incidence in Mid-Ohio Valley residents exposed to PFOA in drinking water due to chemical plant emissions.

Methods: The cohort consisted of adult community residents who resided in contaminated water districts or worked at a local chemical plant. Most participated in a 2005–2006 baseline survey in which serum PFOA was measured. We interviewed the cohort in 2008–2011 to obtain further medical history. Retrospective yearly PFOA serum concentrations were estimated for each participant from 1952 through 2011. Self-reported cancers were validated through medical records and cancer registry review. We estimated the association between cancer and cumulative PFOA serum concentration using proportional hazards models.

Results: Participants (*n* = 32,254) reported 2,507 validated cancers (21 different cancer types). Estimated cumulative serum PFOA concentrations were positively associated with kidney and testicular cancer [hazard ratio (HR) = 1.10; 95% CI: 0.98, 1.24 and HR = 1.34; 95% CI: 1.00, 1.79, respectively, for 1-unit increases in ln-transformed serum PFOA]. Categorical analyses also indicated positive trends with increasing exposures for both cancers: for kidney cancer HRs for increasing exposure quartiles were 1.0, 1.23, 1.48, and 1.58 (linear trend test *p* = 0.18) and for testicular cancer, HRs were 1.0, 1.04, 1.91, 3.17 (linear trend test *p* = 0.04).

Conclusions: PFOA exposure was associated with kidney and testicular cancer in this population. Because this is largely a survivor cohort, findings must be interpreted with caution, especially for highly fatal cancers such as pancreatic and lung cancer.

Citation: Barry V, Winquist A, Steenland K. 2013. Perfluorooctanoic acid (PFOA) exposures and incident cancers among adults living near a chemical plant. Environ Health Perspect 121:1313–1318; http://dx.doi.org/10.1289/ehp.1306615

## Introduction

Perfluorooctanoic acid (PFOA, or C8) is a synthetic chemical used since the late 1940s in manufacturing industrial and household products (Steenland et al. 2010). It is persistent in the environment and has a long human half-life (Lau et al. 2007; Olsen et al. 2007; Seals et al. 2011). PFOA is found at low levels in the serum of most people living in the United States, with higher levels observed in occupationally exposed workers (Calafat et al. 2007; Lau et al. 2007). Exposure sources in the general population are not well established, but likely include diet, drinking water, food packaging, and household products (Lau et al. 2007). PFOA was reported to induce liver, testes, and pancreatic tumors in male rats over a 2-year period (Biegel et al. 2001). However, no evidence was found of hepatocellular, testicular, or pancreatic tumors in male monkeys exposed to PFOA for 26 weeks and observed for 90 days after exposure (Butenhoff et al. 2002). Exposure levels used in the animal studies were higher than human levels typically seen from drinking water or occupational exposure. Because of PFOA’s potential for environmental persistence, long human half-life, and possible toxicity, there is rising concern about whether it might be associated with human cancers (U.S. Environmental Protection Agency 2005, 2006).

The biologic mechanisms by which PFOA caused rat tumors, as well as the pertinence of the animal findings to humans, are unclear. PFOA activation of peroxisome proliferator receptors may cause liver tumors in rats (Kennedy et al. 2004), and also in rats, PFOA-induced increases in serum estradiol levels (Biegel et al. 2001) may have caused testicular tumor growth. It is not known if these processes are relevant to human cancer (DeWitt et al. 2009; Koeffler 2003; Suchanek et al. 2002).

Most previous human studies of the association between PFOA and cancer have been mortality studies of occupationally exposed workers with few cancer deaths. One study followed workers employed at a Minnesota PFOA production plant between 1947 and 1997 (Lundin et al. 2009). These investigators reported some evidence of positive trends for prostate and pancreatic cancer across job categories with increasing PFOA exposure, but estimates were based on only 16 and 13 deaths, respectively.

A second mortality study followed workers who had been employed at any time between 1948 and 2002 at the West Virginia DuPont Washington Works plant considered in the present study (Leonard et al. 2008). These authors reported that kidney cancer mortality was almost doubled among plant workers compared with other regional DuPont workers [standardized mortality ratio (SMR) = 181.0, 95% confidence interval (CI): = 93.5, 316.2]. Steenland and Woskie (2012) recently updated this study and reported a significant increase in kidney cancer mortality with increasing estimated cumulative PFOA serum concentrations based on 12 kidney cancer deaths. SMRs (95% CIs) by increasing exposure quartile were 1.07 (95% CI: 0.02, 3.62), 1.37 (95% CI: 0.28, 3.99), 0 (95% CI: 0, 1.42), and 2.66 (95% CI: 1.15, 5.24) (trend test *p* = 0.02).

There have been two PFOA–cancer incidence studies among general populations: Eriksen et al. (2009) and Bonefeld-Jorgensen et al. (2011). Eriksen et al. (2009) enrolled 57,053 cancer-free Danish adults 50–65 years of age; they measured PFOA plasma concentrations during enrollment and followed participants for approximately 10 years for incident prostate, pancreas, liver, and bladder cancers. Positive associations between PFOA and prostate and pancreatic cancers were reported but were not significant, and no significant linear trends were seen for any of the four cancers. A case–control study of 31 breast cancer cases from the Inuit population (Bonefeld-Jorgensen et al. 2011) reported no relationship between PFOA and breast cancer. The unadjusted odds ratio (OR) was 1.07 (95% CI: 0.88, 1.31). PFOA levels are typically low and widespread in general populations.

The DuPont chemical plant in Washington, West Virginia, began using PFOA in its manufacturing process in 1951. The plant released PFOA into the Ohio River and air beginning in the 1950s, peaking in the 1990s, and decreasing emissions after 2001. PFOA emitted from the plant entered the groundwater, which was the public drinking water source.

In 2001, residents living near the plant filed a class action lawsuit alleging health damage due to PFOA-contaminated drinking water. A pretrial settlement required DuPont to provide funding for an independent community health study called the C8 Health Project (C8 Health Project 2012; Frisbee et al. 2009), and also resulted in the creation of the C8 Science Panel (C8 Science Panel 2012), which was tasked with determining whether there was a probable link between PFOA and disease in the community living near the plant.

The C8 Health Project surveyed Mid-Ohio Valley residents in 2005–2006. The survey collected medical history and also measured serum PFOA concentrations. The median serum PFOA concentration in this population was 28 ng/mL in 2005–2006, compared with 4 ng/mL in the United States overall (Calafat et al. 2007; Steenland et al. 2009).

Using the C8 Health Project cohort in combination with a DuPont worker cohort, the C8 Science Panel conducted subsequent interviews in 2008–2011 to gather disease incidence data. Cancer incidence results from that investigation are reported here.

## Methods

*Data sources and study participants*. The C8 Health Project surveyed 69,030 persons between August 2005 and August 2006. Participants were eligible if they lived, worked, or attended school for ≥ 1 year in one of six contaminated water districts near the plant between 1950 and 3 December 2004. Participants reported demographic and health characteristics and an extensive residential history. Serum was collected for PFOA measurements. The estimated C8 Health Project participation rate was high (81% among current residents ≥ 20 years of age) (Frisbee et al. 2009). A detailed study description has been published previously (Frisbee et al. 2009).

The C8 Science Panel sought to enroll adult C8 Health Project participants in subsequent surveys to study disease incidence, and 74% of the participants ≥ 20 years of age consented to further contact by the C8 Science Panel. Of these, 82% participated in one or two surveys during 2008–2011. The C8 Health Project participants who completed at least one subsequent survey did not differ significantly from the original adult C8 Health Project participants with respect to age, sex, education, water district, or PFOA serum concentrations measured during 2005–2006. They reported demographic information, health-related behaviors, and medical history. In addition, we obtained a list of DuPont workers who formed a cohort that was originally constructed for a mortality study (Leonard et al. 2008; Steenland and Woskie 2012). This DuPont cohort was formed by DuPont and included 6,026 workers who were employed at the Washington, West Virginia, plant for ≥ 1 day between 1 January 1948 and 31 December 2002. Of these, we interviewed 4,391 workers, including 1,890 who were also enrolled in the C8 Health Project.

**Figure 1:**
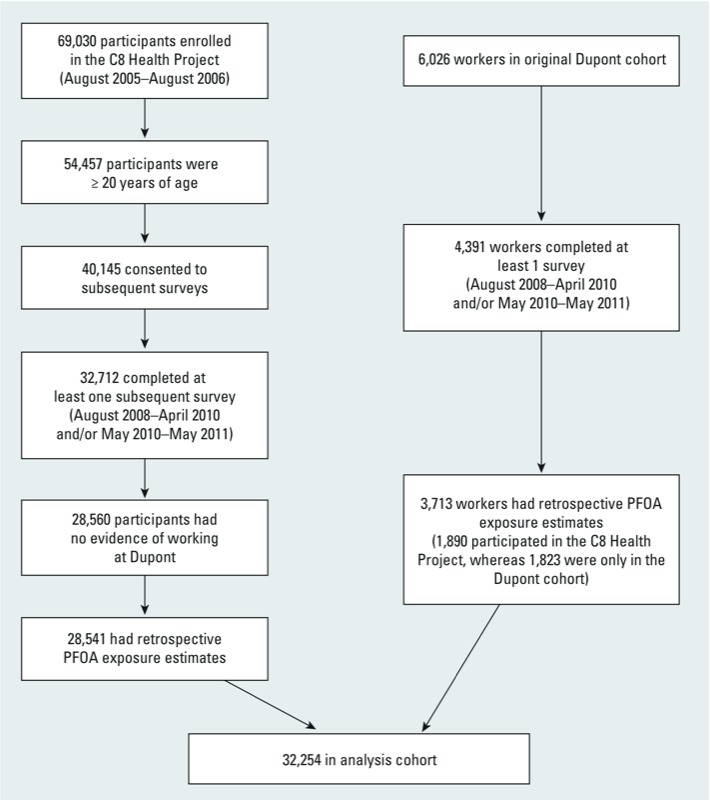
Cohort enrollment.

Figure 1 shows how the analysis cohort was compiled. The analysis included 32,254 persons ≥ 20 years of age, who participated in at least one subsequent survey and had exposure estimates.

All participants gave informed consent to participate, to match personal information to state cancer registries, and to release medical records to study personnel. Medical records were protected in accordance with the Health Insurance Portability and Accountability Act (HIPAA) regulation. The study was approved by the Emory University Institutional Review Board.

*PFOA estimates*. Cumulative PFOA serum concentration estimates were calculated retrospectively for each community participant for each year of life beginning in 1952 or the participant’s birth year, whichever was most recent, through 2011. Estimation procedure details have been published previously (Shin et al. 2011a, 2011b). Estimates were based on historical regional data including the PFOA amounts emitted by the DuPont facility, wind patterns, river flow, and groundwater flow. Exposure estimates took into account each participant’s reported residential history, drinking-water source, tap-water consumption, workplace water consumption, and a PFOA absorption, distribution, metabolism, and excretion model.

The exposure estimates for participants who had ever worked at the DuPont plant took into account occupational exposure they may have received at their specific job. Estimated serum levels over time for workers in different plant jobs were based on over 2,000 PFOA serum measurements taken over time from workers (Woskie et al. 2012). These estimates were used to create a job–exposure matrix to estimate serum levels for workers across time in different jobs and departments. After employment ended, exposure estimates decayed at a rate of 18% per year based on a presumed half-life of 3.5 years (Olsen et al. 2007). These estimates were then combined with estimated serum levels from residential exposure to contaminated drinking water. We estimated combined residential and occupational exposure for 3,713 (84%) of the interviewed workers.

*Cancer data and confirmation process*. Participants were asked “Have you ever been told by a doctor or other health professional that you had cancer or a malignancy of any kind?” Participants reported the cancer type and their age at diagnosis. Those reporting cancer were asked to allow us to review their medical records. For all self-reported cancers, we sought diagnosis validation though medical chart review or Ohio/West Virginia state cancer registry matching.

The Ohio state cancer registry was begun in 1992 and the West Virginia registry in 1993. If a participant who self-reported a cancer type was found in either of the state cancer registries to have that cancer, we confirmed their cancer using the registry. We also sought medical records for participants who reported cancer and who consented for us to do so. Some participants who reported cancer were not identified in the registries (possibly due to living out of state or receiving a cancer diagnosis prior to 1992) and in these cases, we used their medical records to confirm self-reported cancer. Medical records were received from doctors the participant reported were relevant to the specific condition and ranged from primary care physician records to oncologist records. We confirmed cancers if there was sufficient information in the record. This information could include mention of cancer diagnosis, treatments received, ICD-9 [*International Classification of Diseases, Ninth Revision, Clinical Modification* (ICD-9-CM) (National Center for Health Statistics 2006)] and ICD-10 [*International Statistical Classification of Diseases and Related Health Problems, 10th Revision* (World Health Organization 1992)] codes, or specific cancer- or tumor-descriptive characteristics.

*Statistical analysis*. Our main analyses were restricted to validated primary cancers. Participants who reported a cancer that was not validated were excluded from the specific cancer model and thus did not contribute any person-time to the model.

A proportional hazards regression model was run for each cancer type with the cancer as the outcome, time-varying cumulative PFOA serum concentration as the independent variable, and age as the time scale. Participants were followed from the age of 20 years or age in 1952 (the year after the first PFOA emissions), whichever was later, to cancer diagnosis age, last survey age, or death age (if deceased), whichever came first. Each model was adjusted for time-varying smoking, time-varying alcohol consumption, sex, education, and 5-year birth year period. We checked the proportional-hazards assumption for each model by including an exposure × age interaction, and found no violation of the proportional-hazards assumption (all interaction *p*-values > 0.05).

Our primary exposure metric was cumulative PFOA serum concentration (in nanograms per milliliter–years), which was calculated as the sum of all yearly serum concentration estimates up to a given age. We considered models that included the natural log of cumulative PFOA serum concentration as a continuous variable (a test for trend), and models that included categorical variables for cumulative serum concentration quartiles. The log of cumulative serum concentration consistently fit better than the linear untransformed cumulative serum concentration [based on the Akaike information criterion (AIC)], presumably because log transformation diminished the influence of relatively sparse data with very high cumulative exposure. The interpretation of the log cumulative exposure coefficient is that an increase of one unit of log cumulative exposure results in a relative risk (RR) of e^β^ compared with those with one unit less. We also tested for a linear trend in log RRs in categorical analyses by assigning the midpoint to each quartile and conducting a weighted linear regression of the log RRs on these midpoints.

Quartile cut points were calculated among the cumulative PFOA serum concentration estimates for the cancer-specific cases at diagnosis time. We also considered models that lagged cumulative PFOA serum concentration by 10 and 20 years in order to consider scenarios in which cancer could have been caused by exposure further in the past. Here we report the models that lagged cumulative PFOA serum concentration by 10 years. We also ran models limited to community residents who did not work at the plant in order to explore whether results were driven by the high PFOA exposure experienced by workers. Quartile cut points were recalculated for every cancer and population subgroup model.

## Results

*Demographic characteristics*. Table 1 displays descriptive data for the 32,254 participants. Participants were, on average, 53 years of age at the time of their final survey, with male participants slightly older than female (54 years vs. 52 years). Most participants were of white race and were community residents. Eleven percent had ever worked at the DuPont plant. Female participants were more likely to have some college education than were male participants (36% of women vs. 29% of men). Participants who had ever worked at the DuPont plant were more likely to be male and older at the time of interview compared with participants without DuPont work experience (80% vs. 42% and 59 vs. 52 years of age).

**Table 1:** Demographic characteristics of cohort (*n* = 32,254) by community and occupational groups [*n* (%) or mean ± SD].

Characteristic	Entire cohort (*n* = 32,254)	Community group (*n* = 28,541)	Occupational group (*n* = 3,713)
Sex
Male	14,894 (46.2)	11,939 (41.8)	2,955 (79.6)
Female	17,360 (53.8)	16,602 (58.2)	758 (20.4)
Race/ethnicity^*a*^
White, non-Hispanic	31,144 (97.4)	27,860 (97.6)	3,284 (96.1)
Other	815 (2.6)	681 (2.4)	134 (3.9)
Education^*b*^
Less than high school	3,063 (9.5)	3,026 (10.6)	37 (1.0)
High school or certificate of equivalency (GED)	12,971 (40.2)	11,706 (41.0)	1,265 (34.1)
Some college	10,522 (32.6)	9,441 (33.1)	1,081 (29.1)
Bachelor or higher	5,694 (17.7)	4,366 (15.3)	1,328 (35.8)
Mean age at final interview (years)	53.0 ± 15.6	52.2 ± 15.6	59.3 ± 14.1
Mean year of birth	1957 ± 15.6	1958 ± 15.6	1951 ± 14.1
Type of participant
Community only	28,541 (88.5)	28,541 (100.0)	—
Worker only	1,823 (5.7)	—	1,823 (49.1)
Community and worker	1,890 (5.9)	—	1,890 (50.9)
GED, General Education Development test. ^***a***^Race/ethnicity information was missing for 295 participants (all from the occupational group). ^***b***^Education information was missing for 4 participants (2 from the community group and 2 from the occupational group).			

Participants who had worked at the plant had higher PFOA serum levels in 2005–2006 and also had higher estimated annual PFOA serum levels compared with participants who never worked at the plant (Table 2). On average, each participant contributed 33 follow-up years after 20 years of age but estimated serum levels were low prior to 1980.

**Table 2:** Measured and estimated PFOA exposure concentrations (ng/mL) in the cohort (*n* = 32,254).

Cohort	Median (range)
Measured PFOA serum level in 2005–2006
Community (*n* = 28,541)	24.2 (0.25–4,752)
Worker (*n* = 1,881)^*a*^	112.7 (0.25–22,412)
Estimated annual PFOA serum level^*b*^
Community (*n* = 28,541)	19.4 (2.8–9,217)
Worker (*n* = 3,713)	174.4 (5.2–3,683)
^***a***^Workers who did not participate in the C8 Health Project did not have serum levels measured (*n* = 1,823) and other workers were missing measurements (*n* = 9). ^***b***^Community residents were followed for an average of 32 years, and ­workers were followed for an average of 38 years.	

Participants reported 3,589 different cancer diagnoses covering 21 cancer types; 2,507 cancer diagnoses were validated (70%). Table 3 shows the number of cancer diagnoses reported, the number with a received medical record or state cancer registry entry, and the number validated. We obtained a record to review for 88% of self-reported cancers. Reasons for nonvalidation included living in a different state, having a cancer prior to the existence of the two cancer registries, or failing to consent for medical record review. The accuracy of self-reported cancer varied by cancer site. Breast, bladder, kidney, prostate, thyroid, colorectal, lung, leukemia, and lymphoma cancers were more likely to be confirmed compared with other cancer types. Cervical cancer had a low validation rate, possibly due to participants misinterpreting abnormal pap smear results. Cancer was more often validated in DuPont worker participants compared with community residents who never worked at DuPont (75% vs. 69%) (see Supplemental Material, Table S1).

**Table 3:** Number of reported and validated^*a*^ primary cancer cases among the cohort (*n* = 32,254).

Cancer	No. reported	No. reported (had a medical record reviewed or a cancer registry entry)	No. validated [*n* (%)]
Bladder	115	115	111 (96.5)
Brain	33	31	23 (69.7)
Breast	608	600	581 (95.6)
Cervical	383	245	22 (5.7)
Colorectal	311	297	276 (88.7)
Esophagus	21	19	15 (71.4)
Kidney	124	117	113 (91.1)
Leukemia	79	71	69 (87.3)
Liver	18	15	10 (55.6)
Lung	133	124	113 (85.0)
Lymphoma	164	158	142 (86.6)
Melanoma	519	414	245 (47.2)
Oral	35	34	20 (57.1)
Ovarian	87	65	43 (49.4)
Pancreatic	35	31	26 (74.3)
Prostate	515	476	458 (88.9)
Soft tissue	25	19	17 (68.0)
Stomach	29	24	12 (41.4)
Testicular	32	21	19 (59.4)
Thyroid	98	97	87 (88.8)
Uterine	225	173	105 (46.7)
Total	3,589^*b*^	3,146	2,507^*c*^ (69.9)
^***a***^Validated cases were limited to participants who reported the cancer and were subsequently confirmed either by Ohio/West Virginia cancer registry or medical record review; participants reported whether a doctor had ever told them they had a cancer or malignancy of any kind. ^***b***^These 3,589 cancers were self-reported by 3,292 participants; some ­participants reported more than one cancer type. ^***c***^These 2,507 cancers are among 2,361 participants.			

*Exposure–outcome associations*. Table 4 shows adjusted proportional hazards model results for each cancer type based on validated cases only. Thyroid, kidney, and testicular cancer risk increased with an increase in the log of estimated cumulative PFOA serum concentration (Table 4); this association was statistically significant only for testicular cancer at the *p* = 0.05 level. The hazard ratios (HRs) and 95% CIs were similar between models where exposure was unlagged, models where exposure was lagged 10 years, and models where exposure was lagged 20 years (results not shown). The models generally fit slightly better for unlagged exposure compared with 10- and 20-year lagged exposures, as measured by AIC. Results based on all self-reported cancer cases were similar to estimates based on validated cases only (data not shown). The increase in testicular and kidney cancer risk by increasing log of estimated cumulative PFOA serum concentration was stronger in community residents compared with DuPont workers (see Supplemental Material, Table S2). However, the association between thyroid cancer risk and PFOA was positive and significant in DuPont workers but not community residents (see Supplemental Material, Table S2).

**Table 4:** HRs (95% CIs) for the effect of logged estimated cumulative PFOA serum concentration on cancer risk in the cohort (*n* = 32,254).

Cancer^*a*^	No. of cases^*b*^	No lag		10-year lag	
		HR (95% CI)^*c*^	*p*-Value	HR (95% CI)^*c*^	*p*-Value
Bladder	105	1.00 (0.89, 1.12)	0.98	0.98 (0.88, 1.10)	0.77
Brain	17	1.13 (0.84, 1.51)	0.43	1.06 (0.79, 1.41)	0.70
Breast	559	0.94 (0.89, 1.00)	0.05	0.93 (0.88, 0.99)	0.03
Cervical	22	0.89 (0.63, 1.24)	0.48	0.98 (0.69, 1.38)	0.90
Colorectal	264	0.99 (0.92, 1.07)	0.84	0.99 (0.92, 1.07)	0.77
Esophagus	15	0.96 (0.70, 1.32)	0.82	0.97 (0.72, 1.31)	0.84
Kidney	105	1.10 (0.98, 1.24)	0.10	1.09 (0.97, 1.21)	0.15
Leukemia	66	1.01 (0.87, 1.18)	0.88	1.02 (0.88, 1.18)	0.80
Liver	9	0.73 (0.43, 1.23)	0.23	0.74 (0.43, 1.26)	0.26
Lung	108	0.88 (0.78, 1.00)	0.05	0.92 (0.81, 1.04)	0.17
Lymphoma	136	1.01 (0.91, 1.12)	0.88	0.98 (0.88, 1.10)	0.78
Melanoma	241	1.00 (0.92, 1.09)	0.97	1.04 (0.96, 1.13)	0.30
Oral	18	0.89 (0.65, 1.22)	0.46	0.66 (0.43, 1.02)	0.06
Ovarian	43	0.95 (0.76, 1.19)	0.64	0.90 (0.69, 1.16)	0.42
Pancreatic	24	1.00 (0.78, 1.29)	0.99	0.96 (0.75, 1.22)	0.72
Prostate	446	0.99 (0.93, 1.04)	0.63	0.99 (0.94, 1.05)	0.80
Soft tissue	15	0.75 (0.51, 1.10)	0.14	0.72 (0.48, 1.09)	0.12
Stomach	12	0.72 (0.45, 1.14)	0.16	0.77 (0.49, 1.22)	0.27
Testicular	17	1.34 (1.00, 1.79)	0.05	1.28 (0.95, 1.73)	0.10
Thyroid	86	1.10 (0.95, 1.26)	0.20	1.04 (0.89, 1.20)	0.65
Uterine	103	1.05 (0.91, 1.20)	0.53	0.99 (0.86, 1.15)	0.94
^***a***^A proportional hazards regression model was run for each cancer; each model was adjusted for time-varying smoking, time-varying alcohol consumption, sex, education, and stratified by 5-year period of birth year; time began at age 20 years if the person’s 20th birthday was in 1952 or later, otherwise time began at the age the person was in 1952; time ended at the age of cancer diagnosis, age at the last follow-up survey, or age on 31 December 2011, whichever came first. ^***b***^Number of cancer cases used in the regression model (i.e., no missing data for any of the model’s covariates). ^***c***^Per unit of log estimated cumulative PFOA serum concentration (ng/mL).					

Table 5 reports proportional hazards model results for selected cancers using estimated cumulative PFOA serum concentration quartiles. Estimated RRs for kidney cancer and testicular cancer generally increased monotonically across quartiles, while the pattern across thyroid cancer quartiles was less consistent. *p*-Values for linear trend tests of log rate ratios across quartiles of unlagged exposures (using exposure category midpoints, and inverse variance weighting in a no-intercept linear regression model) were 0.25, 0.18, and 0.04 for thyroid, kidney, and testicular cancers, respectively. The *p*-values for thyroid, kidney, and testicular cancer trend tests with a 10-year lag were 0.57, 0.34, and 0.02. When stratified by occupational status, estimated RRs for thyroid cancer increased monotonically across quartiles among DuPont workers, but did not increase monotonically for kidney cancer among DuPont workers (see Supplemental Material, Table S3). Results for the worker cohort are limited by low sample size for cancers of interest.

**Table 5:** HRs (95% CIs) by PFOA quartile^*a*^ for thyroid, kidney, and testicular cancer cases among the cohort (*n* = 32,254).

Cancer	No. of cases^*b*^	Quartile 1 (reference)	Quartile 2	Quartile 3	Quartile 4	*p*-Value^*c*^	*p*-Value^*d*^
Kidney
No lag	105	1.00	1.23 (0.70, 2.17)	1.48 (0.84, 2.60)	1.58 (0.88, 2.84)	0.18	0.10
10-year lag	105	1.00	0.99 (0.53, 1.85)	1.69 (0.93, 3.07)	1.43 (0.76, 2.69)	0.34	0.15
Testes
No lag	17	1.00	1.04 (0.26, 4.22)	1.91 (0.47, 7.75)	3.17 (0.75, 13.45)	0.04	0.05
10-year lag	17	1.00	0.87 (0.15, 4.88)	1.08 (0.20, 5.90)	2.36 (0.41, 13.65)	0.02	0.10
Thyroid
No lag	86	1.00	1.54 (0.77, 3.12)	1.48 (0.74, 2.93)	1.73 (0.85, 3.54)	0.25	0.20
10-year lag	86	1.00	2.06 (0.93, 4.56)	2.02 (0.90, 4.52)	1.51 (0.67, 3.39)	0.57	0.65
^***a***^Quartiles were defined by the estimated cumulative PFOA serum concentration among the thyroid, kidney, or testicular cancer cases at the time of cancer diagnosis. ^***b***^A proportional hazards regression model was run for each cancer; each model was adjusted for time-varying smoking, time-varying alcohol consumption, sex, education, and stratified by 5-year period of birth year. Time began at age 20 years if the person’s 20th birthday was in 1952 or later; otherwise time began at the age the person was in 1952; time ended at the age of cancer diagnosis, age at the last follow-up survey, or age on December 31st 2011, whichever came first. ^***c***^*p*-Value is for linear trend test in the log rate ratios across quartiles; *p*-Values were calculated using exposure category midpoints and inverse variance weighting in a no-intercept linear regression model. ^***d***^*p*-Value is from the continuous log estimated cumulative PFOA serum concentration models.							

Because thyroid cancer is more common in women, perhaps reflecting different mechanisms from men, we ran separate analyses for men and women (24 and 74 cases, respectively). Results were similar in each group (data not shown).

*Sensitivity analyses*. We conducted several sensitivity analyses. We looked back at each participant’s residential history and estimated the time when each participant was first known to have begun living or working in one of the six contaminated water districts, excluding prior time. We then considered survival models that started each person’s time on this “qualifying date,” excluding years before that date. These analyses resulted in slightly less person-time and slightly fewer cancer cases than original analyses; again, results were similar to reported results. HRs for a 1-unit increase in ln-transformed cumulative exposure in relation to thyroid, kidney, and testicular cancers were 1.06 (95% CI: 0.92, 1.23), 1.12 (95% CI: 0.99, 1.26), and 1.37 (95% CI: 0.99, 1.90) for unlagged exposures, and 1.02 (95% CI: 0.87, 1.19), 1.10 (95% CI: 0.98, 1.24), and 1.31 (95% CI: 0.95, 1.81) for exposures lagged by 10 years.

## Discussion

We estimated associations between estimated cumulative PFOA exposures and incident cancers among a group of individuals exposed to PFOA through drinking water or work at the local DuPont chemical plant. Positive associations between PFOA and cancer were found for kidney, testicular, and thyroid cancer.

The positive exposure–response trend for kidney cancer is consistent with a previous DuPont worker mortality analysis, which indicated a positive exposure–response trend for kidney cancer deaths (Steenland and Woskie 2012). Our findings are also in agreement with an ecological study of incident cancer rates in relation to PFOA exposure levels between 1996 and 2005 in five Ohio and eight West Virginia counties (Vieira et al. 2013), which included some cancers diagnosed among participants in the present study population. They reported a significant positive association between kidney cancer and the two highest estimated PFOA serum exposure categories. Finally, the kidney was of *a priori* interest because studies using rats, mice, hamsters, rabbits, and chickens have shown that PFOA is distributed mainly in the kidneys, liver, and serum (Han et al. 2005; Kennedy et al. 2004; Lau et al. 2007).

Testicular cancer was of *a priori* interest because PFOA has been shown to induce testicular tumors in male rats (Biegel et al. 2001) and also to increase estradiol production in male rats, which may increase testicular tumor risk (Biegel et al. 2001). In the ecological study performed by Vieira et al. (2013), estimated PFOA exposures were positively associated with testicular cancer. As noted above, cases included in the ecological study would have partly overlapped with cases diagnosed in our study population.

To our knowledge, there are no reports of an association between PFOA and thyroid cancer from experimental studies of animals or observational studies of human populations. However, there is evidence that PFOA is associated with incident nonmalignant thyroid disease in this population (Winquist and Steenland 2012).

We confirmed self-reported cancers through state cancer registry matching and medical record review. Our cancer validation rates for breast, prostate, lung, and melanoma cancers are similar to those in previous studies, suggesting that breast, prostate, and lung cancers are typically reported accurately, whereas rectal cancer and melanoma of the skin may be reported less accurately (Bergmann et al. 1998; Stavrou et al. 2011). We tried to avoid these problems by grouping self-reported cases of “colon” and “rectal” cancer as “colorectal” cancer cases. Similarly, we did not evaluate non-melanoma skin cancer as an outcome and limited melanoma cases to participants confirmed for melanoma.

Community cohort participants (*n* = 30,431) had to be alive in 2004–2005 to participate in the C8 Health Project, and thus to be eligible for inclusion in our community cohort. Worker cohort participants who were not in the C8 Health Project (1,823) did not have to be alive in 2004–2005 to be included in the study. Nevertheless, because of difficulties in obtaining proxy respondents for deceased target cohort members at time of interview in 2008–2011, most of the participants from both cohorts were alive at the time of their interview in 2008–2011. It is possible that some potentially eligible kidney cancer cases would not have been enrolled or interviewed because they died before 2005, given that the 5-year survival rate for kidney cancer based on 2002–2008 SEER (Surveillance Epidemiology and End Results) data was only 70% (National Cancer Institute 2012). In contrast, cancers with low fatality rates, such as thyroid and testicular cancer, would not be expected to be missing from the study cohort. If cancer cases with higher exposure were more likely to die before they could be enrolled in our cohort, associations with PFOA may be biased toward the null, particularly for highly fatal cancers like pancreatic cancer and lung cancer; consequently our results must be interpreted with caution. On the other hand, associations could be biased away from the null if a disproportionate number of highly exposed cancer cases participated in the study.

This study has several other limitations. PFOA was estimated individually for each year of each participant’s life based on their self-reported residential history, DuPont PFOA emission patterns, and a PFOA absorption, distribution, metabolism, and excretion model. There is likely misclassification in exposure estimates, although we did find good agreement between model-predicted and measured serum levels in 2005–2006 among the C8 Health Project participants who had never worked at the DuPont plant (*r* = 0.67) (Shin et al. 2011b). Misclassification could cause bias if it was differential according to the outcomes evaluated. Nondifferential misclassification is more likely to result in bias toward the null than away from the null, but not always (Armstrong 1998; Steenland et al. 2000). Also, the cancer validation process was implemented only for those who self-reported a cancer. There could have been participants who had a history of cancer but did not report it. However, potential misclassification of cases as noncases would have a smaller impact on the analysis than misclassification of noncases as cases because the number of cases misclassified as noncases is likely small relative to the total number of noncases.

## Conclusion

Previous research on PFOA and cancer has been primarily restricted to animal experiments, mortality studies of male workers with occupational exposure, and community studies of populations with low exposure levels and human studies have been limited by small numbers of cancer cases. The present study estimated RRs of incident cancers in association with cumulative PFOA exposure in a large community with a range of exposure levels. More than 2,500 validated cancers covering 21 different cancer types were included in the analysis, making it one of the largest cohorts ever used to examine PFOA and cancer. Our findings indicate that PFOA exposure was positively associated with kidney and testicular cancer in this Mid-Ohio Valley population. Because this is largely a survivor cohort, results for highly fatal cancers must be interpreted with caution.

## Supplemental Material

(467 KB) PDF
