# Supplementary material for: Perfluorooctanoic Acid (PFOA) Exposures and Incident Cancers among Adults Living Near a Chemical Plant
Source: Environ Health Perspect. 2013 Sep 5;121(11-12):1313–8. doi: 10.1289/ehp.1306615 (PMC3855514; doi:10.1289/ehp.1306615)
Supplement: (467 KB) PDF [file ehp.1306615.s001.508.pdf]

Environmental Health Perspectives doi:10.1289/ehp.1306615

SUPPLEMENTAL MATERIAL

Perfluorooctanoic Acid (PFOA) Exposures and Incident Cancers among Adults Living Near a Chemical Plant

Vaughn Barry, Andrea Winquist, and Kyle Steenland

Table of Contents

Supplemental Material, Table S1: Reported cancer in community and occupational cohorts……………2

Supplemental Material, Table S2: Cancer risk using continuous exposure………...……………………..3

Supplemental Material, Table S3: Cancer risk using quartiles of exposure………………………………5

Supplemental Material, Table S1. Number of reported and validated[a] primary cancer cases among community (n=28,541) and occupational (n=3,713) groups

Group	Community		Occupational	
Cancer	# reported	# validated (% validated)	# reported	# validated (% validated)
Bladder	83	80 (96.4)	32	31 (96.9)
Brain	26	18 (69.2)	7	5 (71.4)
Breast	589	566 (96.1)	19	15 (79.0)
Cervical	369	21 (5.7)	14	1 (7.1)
Colorectal	264	232 (87.9)	47	44 (93.6)
Esophagus	16	12 (75.0)	5	3 (60.0)
Kidney	102	94 (92.2)	22	19 (86.4)
Leukemia	62	55 (88.7)	17	14 (82.4)
Liver	16	9 (56.3)	2	1 (50.0)
Lung	113	97 (85.8)	20	16 (80.0)
Lymphoma	145	126 (86.9)	19	16 (84.2)
Melanoma	444	204 (46.0)	75	41 (54.7)
Oral	32	19 (59.4)	3	1 (33.3)
Ovarian	85	43 (50.6)	2	0 (0)
Pancreatic	26	22 (84.6)	9	4 (44.4)
Prostate	354	322 (91.0)	161	136 (84.5)
Soft Tissue	20	14 (70.0)	5	3 (60.0)
Stomach	26	11 (42.3)	3	1 (33.3)
Testicular	27	17 (63.0)	5	2 (40.0)
Thyroid	87	79 (90.8)	11	8 (72.7)
Uterine	213	98 (46.0)	12	7 (58.3)
TOTAL	3099[b]	2139 (69.0)	490[c]	368 (75.1)

[a]Validated cases were limited to participants who reported the cancer and were subsequently confirmed either by Ohio/West Virginia cancer registry or medical record review; participants reported whether a doctor had ever told them they had a cancer or malignancy of any kind

[b]These 3,099 cancers were self-reported by 2,851 participants; some participants reported more than 1 cancer type

[c]These 490 cancers were self-reported by 441 participants; some participants reported more than 1 cancer type

Supplemental Material, Table S2. Hazard ratios and 95% confidence intervals assessing the effect of logged estimated cumulative PFOA serum concentration on cancer risk in the community (n=28,541) and occupational (n=3,713) groups

		NO LAG			10 YEAR LAG		
		# cases	HR (95% CI)[a]	p-value	# cases	HR (95% CI)[a]	p-value
Cancer[b]	Group						
Bladder	Community	76	0.96 (0.81, 1.14)	0.65	76	0.90 (0.75, 1.09)	0.29
	Occupational	29	0.65 (0.44, 0.95)	0.02	29	0.73 (0.55, 0.98)	0.04
Brain	Community	13	1.14 (0.78, 1.65)	0.50	13	1.02 (0.68, 1.52)	0.94
	Occupational	4	0.82 (0.26, 2.59)	0.74	4	0.73 (0.32, 1.67)	0.46
Breast	Community	546	0.96 (0.90, 1.02)	0.16	546	0.95 (0.89, 1.01)	0.11
	Occupational	13	1.01 (0.59, 1.74)	0.97	13	1.03 (0.59, 1.79)	0.92
Cervical	Community	21	0.94 (0.67, 1.32)	0.74	21	1.02 (0.72, 1.43)	0.92
	Occupational	1	---	---	1	---	---
Colorectal	Community	223	0.98 (0.89, 1.08)	0.75	223	0.98 (0.89, 1.09)	0.77
	Occupational	41	1.12 (0.81, 1.54)	0.50	41	1.08 (0.84, 1.39)	0.55
Esophagus	Community	12	1.00 (0.66, 1.51)	0.99	12	1.01 (0.67, 1.52)	0.96
	Occupational	3	1.42 (0.21, 9.74)	0.72	3	1.17 (0.19, 7.36)	0.86
Kidney	Community	87	1.14 (0.99, 1.32)	0.07	87	1.11 (0.96, 1.29)	0.17
	Occupational	18	0.95 (0.59, 1.52)	0.82	18	0.99 (0.67, 1.46)	0.97
Leukemia	Community	53	0.92 (0.76, 1.13)	0.43	53	0.92 (0.75, 1.13)	0.41
	Occupational	13	1.30 (0.73, 2.33)	0.37	13	1.30 (0.78, 2.18)	0.31
Liver	Community	8	0.62 (0.29, 1.29)	0.20	8	0.53 (0.21, 1.34)	0.18
	Occupational	1	---	---	1	---	---
Lung	Community	95	0.85 (0.73, 1.00)	0.05	95	0.89 (0.76, 1.05)	0.17
	Occupational	13	0.87 (0.51, 1.47)	0.59	13	1.04 (0.68, 1.58)	0.86
Lymphoma	Community	121	1.05 (0.92, 1.19)	0.48	121	1.02 (0.89, 1.17)	0.80
	Occupational	15	1.24 (0.72, 2.14)	0.45	15	1.10 (0.73, 1.65)	0.66
Melanoma	Community	200	0.99 (0.89, 1.10)	0.82	200	1.02 (0.92, 1.14)	0.66
	Occupational	41	0.80 (0.59, 1.08)	0.15	41	0.93 (0.73, 1.18)	0.53
Oral	Community	17	0.96 (0.65, 1.40)	0.82	17	0.77 (0.47, 1.27)	0.31
	Occupational	1	---	---	1	0.70 (0.19, 2.62)	0.60
Ovarian	Community	43	1.00 (0.79, 1.25)	0.97	43	0.94 (0.73, 1.22)	0.66
	Occupational	0	---	---	0	---	---
Pancreatic	Community	21	1.06 (0.79, 1.43)	0.68	21	0.98 (0.72, 1.34)	0.92
	Occupational	3	0.98 (0.21, 4.65)	0.98	3	1.14 (0.33, 3.89)	0.84
Prostate	Community	317	0.97 (0.90, 1.05)	0.50	317	0.98 (0.90, 1.06)	0.58
	Occupational	129	0.94 (0.77, 1.17)	0.59	129	0.98 (0.83, 1.16)	0.83
Soft Tissue	Community	13	0.68 (0.40, 1.14)	0.14	13	0.64 (0.36, 1.13)	0.12
	Occupational	2	1.20 (0.30, 4.76)	0.80	2	0.91 (0.25, 3.33)	0.89
Stomach	Community	11	0.70 (0.40, 1.23)	0.22	11	0.74 (0.41, 1.31)	0.30
	Occupational	1	---	---	1	---	---
Testicular	Community	15	1.73 (1.24, 2.40)	0.01	15	1.53 (1.09, 2.15)	0.01
	Occupational	2	0.85 (0.04, 19.7)	0.92	2	1.61 (0.21, 12.20)	0.65

		NO LAG			10 YEAR LAG		
		# cases	HR (95% CI)[a]	p-value	# cases	HR (95% CI) [a]	p-value
Thyroid	Community	78	1.04 (0.89, 1.23)	0.61	78	1.00 (0.84, 1.20)	0.96
	Occupational	8	1.93 (1.00, 3.71)	0.05	8	1.12 (0.61, 2.05)	0.71
Uterine	Community	96	1.02 (0.88, 1.18)	0.79	96	0.99 (0.84, 1.16)	0.88
	Occupational	7	1.05 (0.56, 1.97)	0.88	7	0.96 (0.42, 2.18)	0.92

[a] per unit of log estimated cumulative PFOA serum concentration

[b] A proportional hazards regression model was run for each cancer. Each model was adjusted for time-dependent smoking, time-dependent alcohol consumption, gender, education, and stratified by 5-year period of birth year. Time began at age 20 if the person's 20th birthday was in 1952 or later. Otherwise time began at the age the person was in 1952. Time ended at age of cancer diagnosis, age at last follow-up survey, or age on December 31st 2011, whichever came first.

--- model did not converge

Supplemental Material, Table S3. Hazard ratios and 95% confidence intervals by PFOA quartile[a] for thyroid, kidney, and testicular cancer cases among the community (n=28,541) and occupational (n=3,713) groups

			Hazard Ratio (95% CI)[b]				
Group	Cancer	#cases	Quartile 1 (Reference)	Quartile 2	Quartile 3	Quartile 4	p-value[c]
Community	Kidney–no lag	87	1.00	1.34 (0.71, 2.52)	1.95 (1.03, 3.70)	2.04 (1.07, 3.88)	0.20
	Kidney –10 yr lag	87	1.00	0.94 (0.45, 1.99)	1.08 (0.52, 2.25)	1.50 (0.72, 3.13)	0.02
Occupational	Kidney–no lag	18	1.00	0.84 (0.21, 3.4)	4.20 (1.07, 16.44)	0.83 (0.20, 3.55)	0.54
	Kidney –10 yr lag	18	1.00	1.22 (0.28, 5.3)	3.27 (0.76, 14.10)	0.99 (0.21, 4.68)	0.42
Community	Testicular–no lag	15	1.00	0.80 (0.16, 3.97)	3.07 (0.61, 15.36)	5.80 (0.97, 34.58)	0.05
	Testicular–10 yr lag	15	1.00	0.98 (0.13, 7.14)	1.54 (0.19, 12.21)	4.66 (0.52, 41.63)	0.02
Occupational	Testicular–no lag	2	---	---	---	---	---
	Testicular–10 yr lag	2	---	---	---	---	---
Community	Thyroid–no lag	78	1.00	1.54 (0.73, 3.26)	1.71 (0.81, 3.59)	1.40 (0.66, 2.97)	0.46
	Thyroid–10 yr lag	78	1.00	2.09 (0.91, 4.82)	1.92 (0.82, 4.50)	1.42 (0.60, 3.37)	0.56
Occupational	Thyroid–no lag	8	1.00	4.64 (0.42, 50.8)	9.70 (0.67, 141.2)	14.72 (0.85, 253.9)	0.04
	Thyroid–10 yr lag	8	1.00	1.65 (0.09, 31.5)	4.52 (0.10, 198.4)	5.85 (0.13, 257.1)	0.01

[a] *nce intervals by PFOA quartile* so that they now meet the supplemental material formatting requirements.e using devices to readQuartiles were defined by the estimated cumulative PFOA serum concentration among the thyroid, kidney, or testicular cancer cases at the time of cancer diagnosis

[b] A proportional hazards regression model was run for each cancer. Each model was adjusted for time-dependent smoking, time-dependent alcohol consumption, gender, education, and stratified by 5-year period of birth year. Time began at age 20 if the person's 20th birthday was in 1952 or later. Otherwise time began at the age the person was in 1952. Time ended at age of cancer diagnosis, age at last follow-up survey, or age on December 31st 2011, whichever came first.

[c] P-value is for linear trend test in log rate ratios across quartiles. P-values were calculated using exposure category mid-points and inverse variance weighting in a no-intercept linear regression model.

--- Not enough cases for quartile analysis
